# Supplementary material for: Protamine 2 deficiency results in Septin 12 abnormalities
Source: Front Cell Dev Biol. 2024 Oct 25;12:1447630. doi: 10.3389/fcell.2024.1447630 (PMC11543461; doi:10.3389/fcell.2024.1447630)
Supplement: Supplementary file 1 [file DataSheet1.PDF]

Supplemental material

Protamine 2 Deficiency Results In Septin 12 Abnormalities

Ondrej Sanovec[1,2+], Michaela Frolikova[1+], Veronika Kraus[1], Jana Vondrakova[1], Maryam Qasemi[1], Daniela Spevakova[1], Ondrej Simonik[1], Lindsay Moritz[3], Drew Lewis Caswell[4], Frantisek Liska[5], Lukas Ded[1], Jiri Cerny[6], Tomer Avidor-Reiss[4,7], Saher Sue Hammoud[3], Hubert Schorle[8], Pavla Postlerova[1], Klaus Steger[9] and Katerina Komrskova[1,10,*]

[1]Laboratory of Reproductive Biology, Institute of Biotechnology, Czech Academy of Sciences, BIOCEV, Vestec, Czech Republic.
[2]Department of Physiology, Faculty of Science, Charles University, Prague, Czech Republic.
[3]Department of Human Genetics, University of Michigan, Ann Arbor, MI, United States.
[4]Department of Biological Sciences, College of Natural Sciences and Mathematics, University of Toledo, Toledo, OH, United States.
[5]Institute of Biology and Medical Genetics, First Faculty of Medicine, Charles University and General University Hospital in Prague, Prague 2, Czech Republic.
[6]Laboratory of Structural Bioinformatics of Proteins, Institute of Biotechnology Czech Academy of Sciences, BIOCEV, Vestec, Czech Republic.
[7]Department of Urology, College of Medicine and Life Sciences, University of Toledo, Toledo, OH, United States.
[8]Department of Developmental Pathology, Institute of Pathology, University Hospital Bonn, Bonn, Germany.
[9]Clinic of Urology, Paediatric Urology and Andrology, Molecular Andrology, Justus Liebig University of Giessen, Giessen, Germany.
[10]Department of Zoology, Faculty of Science, Charles University, Prague, Czech Republic.

+ These authors contributed equally
* Corresponding author

Correspondence to Katerina Komrskova: katerina.komrskova@ibt.cas.cz.

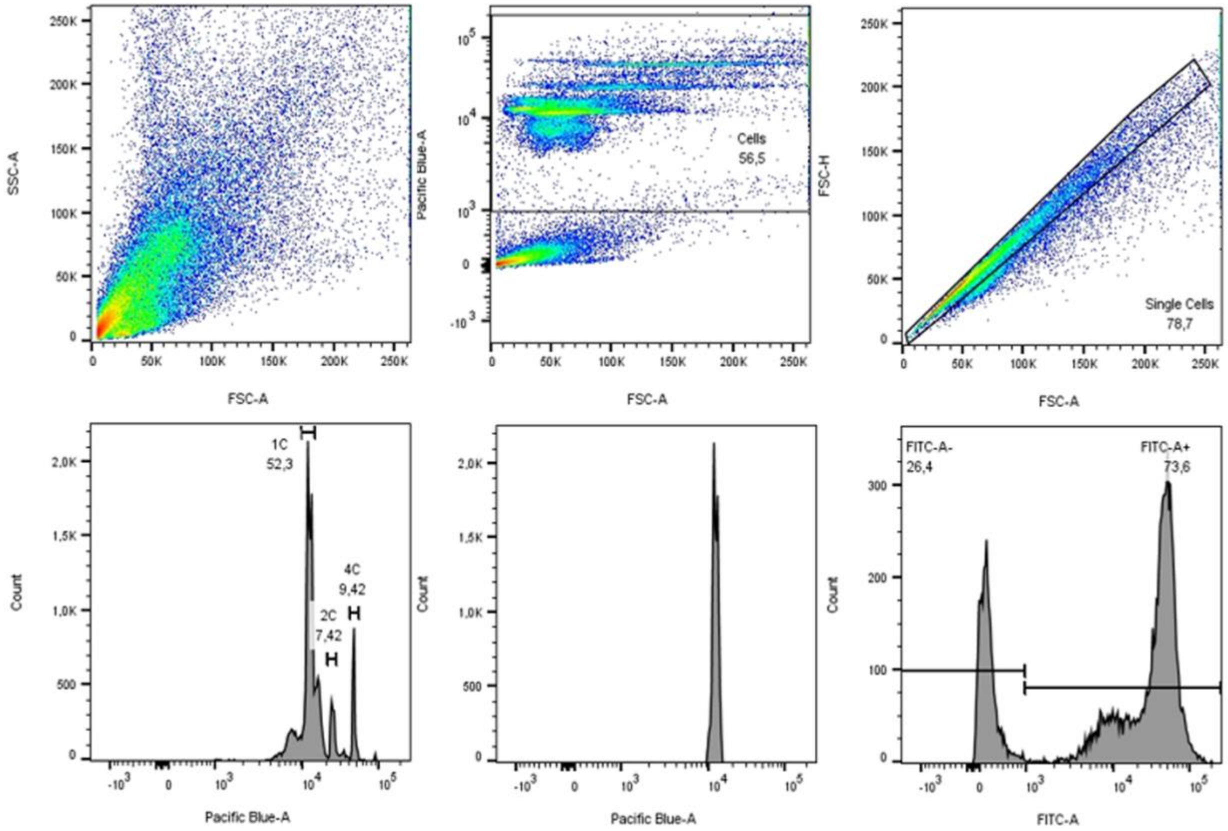

Supplementary Figure S1: Gating strategy of FCM. *Analysis of testicular cell suspension stained with DAPI and antibodies against histone modifications.*

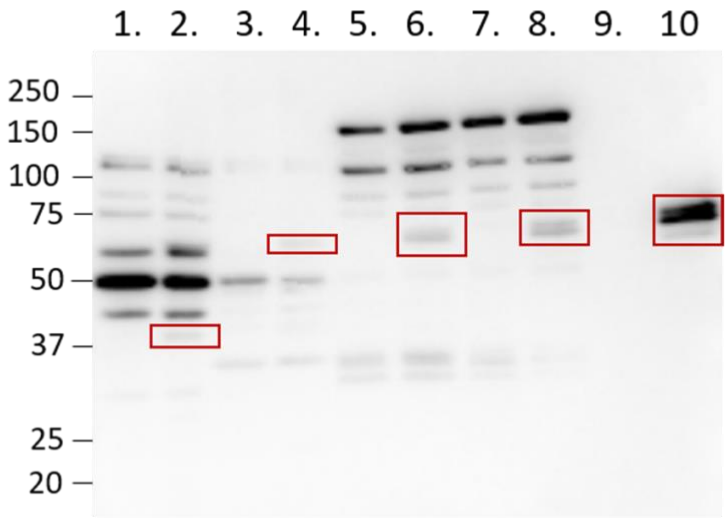

Supplementary Figure S2: Western blot validation of anti-Septin12 antibody*. Subcellular fractions of non-transfected and transfected HEK293T/17 cells. HEK93T/17 cells were transfected with mouse Septin12-GFP plasmid, red boxes mark positive signals. 1 Non-transfected cells cytoplasmic proteins; 2 Septin 12 Transfected cells cytoplasmic proteins; 3 Non-transfected cells membrane proteins; 4 Septin12 transfected cells membrane proteins; 5 Non-transfected cells nuclear protein; 6 Septin12 transfected cells nuclear protein, 7 Non-transfected cells chromatin-bound nuclear proteins; 8 Septin12 transfected cells chromatin-bound nuclear proteins; 9 Non-transfected cells Tubulin/mitochondria protein fraction; 10 Septin12 transfected cells Tubulin/mitochondria protein fraction.*

	WT (n=51)	Prm2$^{-/-}$ (n=76)
Mean signal intensity	15.92 ± 6.46	11.00 ± 3.13
Maximum signal intensity	51.36 ± 21.07	33.62 ± 10.22
Average length	0.47 ± 0.09 µm	0.48 ± 0.10 µm
Average width	0.74 ± 0.20 µm	0.81 ± 0.15 µm

Supplementary Figure S3: CEP135 parameters. *Mean and maximum signal intensity of CEP135 staining and average length and width of centrioles in WT and Prm2$^{-/-}$ caudal sperm.*

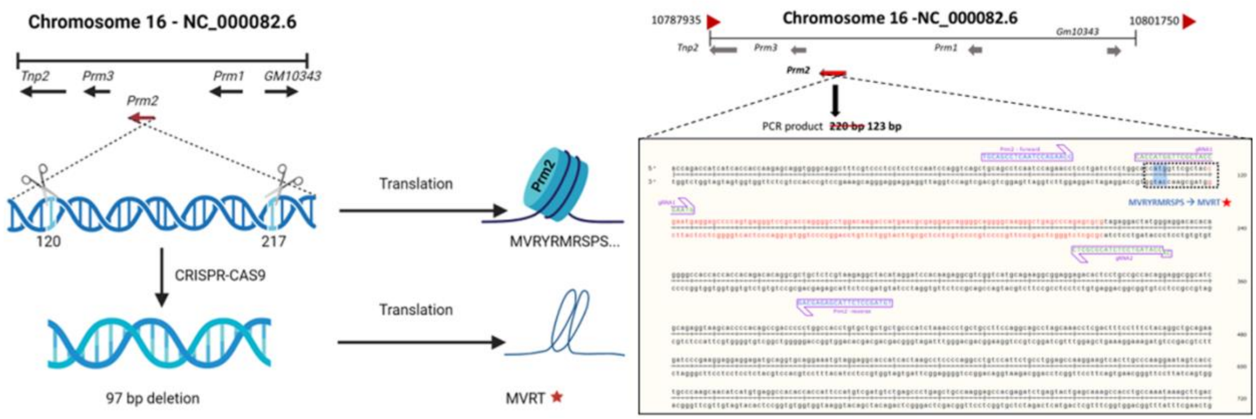

Supplementary Figure S4: Graphical description of mouse model used in this study. ***(A)*** *97 bd deletion was introduced in exon 1 of Prm2 gene, resulting in premature stop codon occurrence.* ***(B)*** *Annotation of the knockout mouse line based on the sequencing. The deleted sequence is in red. gRNA sequences used in CRISPR-CAS9 are shown in green, primer sequences used for genotyping are shown in blue. Asterisk mark stop codon.*

Figure	Number of animals		Biological material
	WT	$Prm2^{-/-}$	
1A-C	5	5	whole testis from each mouse
1D	8	8	whole testis from each mouse
2B,F	3	3	200 sperm from each mouse
2C-E	1	1	106 WT and 176 KO sperm
4A,B	5	5	whole testis from each mouse
4C	8	8	whole testis from each mouse
4D,E	5	5	15 µg/ml of sperm protein lysate
4F	3	3	$5x10^6$ sperm from each animal
5B,C	3	3	13 WT and 11 KO sperm in total
6A-E	1	1	51 WT and 76 KO sperm in total
7A,D	3	3	whole testis from each mouse
7E	8	8	whole testis from each mouse
7F	3	3	sections of testis parts from each mouse

***Supplementary Figure S5: The number of animals used in the study**. This table summarizes the number, type, and amount of biological material used in each relevant experiment.*
